# Strong convergence of an extragradient-type algorithm for the multiple-sets split equality problem

**DOI:** 10.1186/s13660-017-1326-y

**Published:** 2017-02-28

**Authors:** Ying Zhao, Luoyi Shi

**Affiliations:** Department of Mathematics, Tianjin Polytechnic University, Tianjin, 300387 P.R. China

**Keywords:** strong convergence, extragradient-type, multiple-sets split equality problem

## Abstract

This paper introduces a new extragradient-type method to solve the multiple-sets split equality problem (MSSEP). Under some suitable conditions, the strong convergence of an algorithm can be verified in the infinite-dimensional Hilbert spaces. Moreover, several numerical results are given to show the effectiveness of our algorithm.

## Introduction

The split feasibility problem (SFP) was first presented by Censor *et al.* [1]; it is an inverse problem that arises in medical image reconstruction, phase retrieval, radiation therapy treatment, signal processing *etc*. The SFP can be mathematically characterized by finding a point *x* that satisfies the property

1.1 $$\begin{aligned} x\in{C},\quad Ax\in{Q}, \end{aligned}$$

if such a point exists, where *C* and *Q* are nonempty closed convex subsets of Hilbert spaces $H_{1}$ and $H_{2}$, respectively, and $A:H_{1}\rightarrow{H_{2}}$ is a bounded and linear operator.

There are various algorithms proposed to solve the SFP, see [2–4] and the references therein. In particular, Byrne [5, 6] introduced the CQ-algorithm motivated by the idea of an iterative scheme of fixed point theory. Moreover, Censor *et al.* [7] introduced an extension upon the form of SFP in 2005 with an intersection of a family of closed and convex sets instead of the convex set *C*, which is the original of the multiple-sets split feasibility problem (MSSFP).

Subsequently, an important extension, which goes by the name of split equality problem (SEP), was made by Moudafi [8]. It can be mathematically characterized by finding points $x\in{C}$ and $y\in{Q}$ that satisfy the property

1.2 $$\begin{aligned} Ax=By, \end{aligned}$$

if such points exist, where *C* and *Q* are nonempty closed convex subsets of Hilbert spaces $H_{1}$ and $H_{2}$, respectively, $H_{3}$ is also a Hilbert space, $A:H_{1}\rightarrow{H_{3}}$ and $B:H_{2}\rightarrow{H_{3}}$ are two bounded and linear operators. When $B=I$, the SEP reduces to SFP. For more information about the methods for solving SEP, see [9, 10].

This paper considers the multiple-sets split equality problem (MSSEP) which generalizes the MSSFP and SEP and can be mathematically characterized by finding points *x* and *y* that satisfy the property

1.3 $$\begin{aligned} x\in{\bigcap_{i=1}^{t}}C_{i} \quad\mbox{and}\quad y\in{\bigcap_{j=1}^{r}}Q_{j} \quad\mbox{such that } Ax=By, \end{aligned}$$

where $r, t$ are positive integers, $\{C_{i}\}_{i=1}^{t}\in{H_{1}}$ and $\{ Q_{j}\}_{j=1} ^{r}\in{H_{2}}$ are nonempty, closed and convex subsets of Hilbert spaces $H_{1}$ and $H_{2}$, respectively, $H_{3}$ is also a Hilbert space, $A:H_{1}\rightarrow{H_{3}}$, $B:H_{2}\rightarrow{H_{3}}$ are two bounded and linear operators. Obviously, if $B=I$, the MSSEP is just right MSSFP; if $t=r=1$, the MSSEP changes into the SEP. Moreover, when $B=I$ and $t=r=1$, the MSSEP reduces to the SFP.

One of the most important methods for computing the solution of a variational inequality and showing the quick convergence is an extragradient algorithm, which was first introduced by Korpelevich [11]. Moreover, this method was applied for finding a common element of the set of solutions for a variational inequality and the set of fixed points of a nonexpansive mapping, see Nadezhkina *et al.* [12]. Subsequently, Ceng *et al.* in [13] presented an extragradient method, and Yao *et al.* in [14] proposed a subgradient extragradient method to solve the SFP. However, all these methods to solve the problem have only weak convergence in a Hilbert space. On the other hand, a variant extragradient-type method and a subgradient extragradient method introduced by Censor *et al.* [15, 16] possess strong convergence for solving the variational inequality.

Motivated and inspired by the above works, we introduce an extragradient-type method to solve the MSSEP in this paper. Under some suitable conditions, the strong convergence of an algorithm can be verified in the infinite-dimensional Hilbert spaces. Finally, several numerical results are given to show the feasibility of our algorithm.

## Preliminaries

Let *H* be a real Hilbert space whose inner product and norm are denoted by $\langle\cdot,\cdot\rangle$ and $\Vert \cdot \Vert $, respectively. Let *I* denote the identity operator on *H*.

Next, we recall several definitions and basic results that will be available later.

### Definition 2.1

A mapping $T:H\rightarrow{H}$ goes by the name of

(i) nonexpansive if
  $$\Vert Tx-Ty\Vert \leq \Vert x-y\Vert , \quad \forall x,y\in{H}; $$
(ii) firmly nonexpansive if
  $$\Vert Tx-Ty\Vert \leq\langle{x-y,Tx-Ty}\rangle,\quad \forall x,y\in{H}; $$
(iii) contractive on *x* if there exists $0<\alpha<1$ such that
  $$\Vert Tx-Ty\Vert \leq\alpha \Vert x-y\Vert ,\quad \forall x,y\in{H}; $$
(iv) monotone if
  $$\langle{Tx-Ty,x-y}\rangle\geq0,\quad \forall x,y\in{H}; $$
(v) *β*-inverse strongly monotone if there exists $\beta>0$ such that
  $$\langle{Tx-Ty,x-y}\rangle\geq\beta \Vert Tx-Ty\Vert ^{2},\quad \forall x,y\in{H}. $$

The following properties of an orthogonal projection operator were introduced by Bauschke *et al.* in [17], and they will be powerful tools in our analysis.

### Proposition 2.2

[17]

*Let* $P_{C}$ *be a mapping from* *H* *onto a closed*, *convex and nonempty subset* *C* *of* *H* *if*

$$P_{C}(x)=\arg\min_{y\in{C}} \Vert x-y\Vert ,\quad \forall x\in{H}, $$

*then* $P_{C}$ *is called an orthogonal projection from* *H* *onto* *C*. *Furthermore*, *for any* $x,y\in{H}$ *and* $z\in{C}$,

(i) $\langle{x-P_{C}x,z-P_{C}x}\rangle\leq0$;
(ii) $\Vert P_{C}x-P_{C}y\Vert ^{2}\leq\langle{P_{C}x-P_{C}y,x-y}\rangle$;
(iii) $\Vert P_{C}x-z\Vert ^{2}\leq \Vert x-z\Vert ^{2}-\Vert P_{C}x-x\Vert ^{2}$.

The following lemmas provide the main mathematical results in the sequel.

### Lemma 2.3

[18]

*Let* *C* *be a nonempty closed convex subset of a real Hilbert space* *H*, *let* $T:C\rightarrow{H}$ *be* *α*-*inverse strongly monotone*, *and let* $r>0$ *be a constant*. *Then*, *for any* $x,y\in{C}$,

$$\bigl\Vert (I-rT)x-(I-rT)y\bigr\Vert ^{2}\leq \Vert x-y\Vert ^{2}+r(r-2\alpha)\bigl\Vert T(x)-T(y)\bigr\Vert ^{2}. $$

Moreover, when $0< r<2\alpha$, $I-rT$ is nonexpansive.

### Lemma 2.4

[19]

*Let* $\{x^{k}\}$ *and* $\{y^{k}\}$ *be bounded sequences in a Hilbert space* *H*, *and let* $\{\beta_{k}\}$ *be a sequence in* $[0,1]$ *which satisfies the condition* $0<\lim\inf_{k\rightarrow\infty}\beta_{k}\leq{\limsup_{k\rightarrow\infty}\beta_{k}}<1$. *Suppose that* $x^{k+1}=(1-\beta_{k})y^{k}+\beta_{k}x^{k}$ *for all* $k\geq0$ *and* $\lim\sup_{k\rightarrow\infty}(\Vert y^{k+1}-y^{k}\Vert -\Vert x^{k+1}-x^{k}\Vert )\leq0$. *Then* $\lim_{k\rightarrow\infty} \Vert y^{k}-x^{k}\Vert =0$.

The lemma below will be a powerful tool in our analysis.

### Lemma 2.5

[20]

*Let* $\{a_{k}\}$ *be a sequence of nonnegative real numbers satisfying the condition* $a_{k+1}\leq(1-m_{k})a_{k}+m_{k}\delta_{k}, \forall{k\geq0,}$ *where* $\{m_{k}\}$, $\{\delta_{k}\}$ *are sequences of real numbers such that*

(i) $\{m_{k}\}\in[0,1]$ *and* $\sum_{k=0}^{\infty}{m_{k}}=\infty$ *or*, *equivalently*,
  $$\prod_{k=0}^{\infty}(1-m_{k})=\lim _{k\rightarrow\infty}\prod_{j=0}^{k}(1-m_{j})=0; $$
(ii) $\lim\sup_{k\rightarrow\infty}\delta_{k}\leq0$ *or*
(ii)’ $\sum_{k=0}^{\infty}\delta_{k}m_{k}$ *is convergent*. *Then* $\lim_{k\rightarrow\infty}a_{k}=0$.

## Main results

In this section, we propose a formal statement of our present algorithm. Review the multiple-sets split equality problem (MSSEP), without loss of generality, suppose $t>r$ in (1.3) and define $Q_{r+1}=Q_{r+2}=\cdots =Q_{t}=H_{2}$. Hence, MSSEP (1.3) is equivalent to the following problem:

3.1 $$\begin{aligned} \mbox{find}\quad x\in{\bigcap_{i=1}^{t}}C_{i}\quad \mbox{and}\quad y\in{\bigcap_{j=1}^{t}}Q_{j} \quad\mbox{such that } Ax=By. \end{aligned}$$

Moreover, set $S_{i}=C_{i}\times{Q_{i}}\in{H}=H_{1}\times{H_{2}}\ (i=1,2,\ldots ,t)$, $S={\bigcap_{i=1}^{t}}S_{i}$, $G=[A,-B]:H\rightarrow{H_{3}}$, the adjoint operator of *G* is denoted by $G^{*}$, then the original problem (3.1) reduces to

3.2 $$\begin{aligned} \mbox{finding}\quad w=(x,y)\in{S}\quad \mbox{such that } Gw=0. \end{aligned}$$

### Theorem 3.1

*Let* $\Omega\neq\emptyset$ *be the solution set of MSSEP* (3.2). *For an arbitrary initial point* $w_{0}\in{S}$, *the iterative sequence* $\{w_{n}\}$ *can be given as follows*:

3.3 $$\begin{aligned} \textstyle\begin{cases} v_{n}=P_{S}\{(1-\alpha_{n})w_{n}-\gamma_{n}G^{*}Gw_{n}\},\\ w_{n+1}=P_{S}\{w_{n}-\mu_{n}G^{*}Gv_{n}+\lambda_{n}(v_{n}-w_{n})\}, \end{cases}\displaystyle \end{aligned}$$

*where* $\{\alpha_{n}\}_{n=0}^{\infty}$ *is a sequence in* $[0,1]$ *such that* $\lim_{n\rightarrow\infty}\alpha_{n}=0, and \sum_{n=1}^{\infty}\alpha _{n}=\infty$, *and* $\{\gamma_{n}\}_{n=0}^{\infty}$, $\{\lambda_{n}\}_{n=0}^{\infty}$, $\{\mu_{n}\}_{n=0}^{\infty}$ *are sequences in* *H* *satisfying the following conditions*:

3.4 $$\begin{aligned} \textstyle\begin{cases} {\gamma_{n}}\in{(0,\frac{2}{\rho(G^{*}G)})}, \qquad\lim_{n\rightarrow\infty }(\gamma_{n+1}-\gamma_{n})=0;\\ \lambda_{n}\in{(0,1)},\qquad \lim_{n\rightarrow\infty}(\lambda_{n+1}-\lambda _{n})=0;\\ {\mu_{n}}\leq\frac{2}{\rho(G^{*}G)}\lambda_{n}, \qquad\lim_{n\rightarrow\infty}(\mu _{n+1}-\mu_{n})=0;\\ \sum_{n=1}^{\infty}(\frac{\gamma_{n}}{\lambda_{n}})< \infty. \end{cases}\displaystyle \end{aligned}$$

*Then* $\{w_{n}\}$ *converges strongly to a solution of MSSEP* (3.2).

### Proof

In view of the property of the projection, we infer $\hat{w}=P_{S}(\hat {w}-tG^{*}G\hat{w})$ for any $t>0$. Further, from the condition in (3.4), we get that ${\mu_{n}}\leq\frac{2}{\rho(G^{*}G)}\lambda_{n}$, it follows that $I-\frac{\mu_{n}}{\lambda_{n}}G^{*}G$ is nonexpansive. Hence,

3.5 $$\begin{aligned} & \Vert w_{n+1}-\hat{w}\Vert \\ &\quad=\bigl\Vert P_{S}\bigl\{ w_{n}-\mu_{n}G^{*}Gv_{n}+ \lambda_{n}(v_{n}-w_{n})\bigr\} -P_{S} \bigl\{ \hat {w}-tG^{*}G\hat{w}\bigr\} \bigr\Vert \\ &\quad=\biggl\Vert P_{S}\biggl\{ (1-\lambda_{n})w_{n}+ \lambda_{n}\biggl(I-\frac{\mu_{n}}{\lambda _{n}}G^{*}G\biggr)v_{n}\biggr\} -P_{S}\biggl\{ (1-\lambda_{n})\hat{w} +\lambda_{n}\biggl(I-\frac{\mu_{n}}{\lambda_{n}}G^{*}G\biggr)\hat{w}\biggr\} \biggr\Vert \\ &\quad\leq(1-\lambda_{n})\Vert w_{n}-\hat{w}\Vert + \lambda_{n}\biggl\Vert \biggl(I-\frac{\mu_{n}}{\lambda _{n}}G^{*}G \biggr)v_{n}-\biggl(I-\frac{\mu_{n}}{\lambda_{n}}G^{*}G\biggr)\hat{w}\biggr\Vert \\ &\quad \leq(1-\lambda_{n})\Vert w_{n}-\hat{w}\Vert + \lambda_{n}\Vert v_{n}-\hat{w}\Vert . \end{aligned}$$

Since $\alpha_{n}\rightarrow{0}$ as $n\rightarrow\infty$ and from the condition in (3.4), ${\gamma_{n}}\in{(0,\frac{2}{\rho(G^{*}G)})}$, it follows that $\alpha_{n}\leq{1-\frac{\gamma_{n}\rho(G^{*}G)}{2}}$ as $n\rightarrow\infty$, that is, $\frac{\gamma_{n}}{1-\alpha_{n}}\in{(0,\frac {2}{\rho{(G^{*}G)}})}$. We deduce that

3.6 $$\begin{aligned} & \Vert v_{n}-\hat{w}\Vert \\ &\quad=\bigl\Vert P_{S}\bigl\{ (1-\alpha_{n})w_{n}- \gamma_{n}G^{*}Gw_{n}\bigr\} -P_{S}\bigl(\hat{w}-tG^{*}G \hat {w}\bigr)\bigr\Vert \\ &\quad\leq(1-\alpha_{n}) \biggl(w_{n}-\frac{\gamma_{n}}{1-\alpha_{n}}G^{*}Gw_{n} \biggr)-\biggl\{ \alpha _{n}\hat{w}+(1-\alpha_{n}) \biggl( \hat{w}-\frac{\gamma_{n}}{1-\alpha_{n}}G^{*}G\hat{w}\biggr)\biggr\} \\ &\quad\leq\biggl\Vert -\alpha_{n}\hat{w}+(1-\alpha_{n}) \biggl[w_{n}-\frac{\gamma_{n}}{1-\alpha _{n}}G^{*}Gw_{n}-\hat{w}+ \frac{\gamma_{n}}{1-\alpha_{n}}G^{*}G\hat{w}\biggr]\biggr\Vert , \end{aligned}$$

which is equivalent to

3.7 $$\begin{aligned} \Vert v_{n}-\hat{w}\Vert \leq\alpha_{n}\Vert {-\hat{w}} \Vert +(1-\alpha_{n})\Vert w_{n}-\hat{w}\Vert . \end{aligned}$$

Substituting (3.7) in (3.5), we obtain

$$\begin{aligned} \Vert w_{n}-\hat{w}\Vert &\leq(1-\lambda_{n})\Vert w_{n}-\hat{w}\Vert + \lambda_{n}\bigl(\alpha_{n}\Vert {-\hat{w}}\Vert +(1- \alpha_{n})\Vert w_{n}-\hat{w}\Vert \bigr) \\ &\leq(1-\lambda_{n}\alpha_{n})\Vert w_{n}- \hat{w}\Vert +\lambda_{n}\alpha_{n}\Vert {-\hat{w}}\Vert \\ &\leq\max\bigl\{ \Vert w_{n}-\hat{w}\Vert ,\Vert {-\hat{w}}\Vert \bigr\} . \end{aligned}$$

By induction,

$$\Vert w_{n}-\hat{w}\Vert \leq{\max\bigl\{ \Vert w_{0}- \hat{w}\Vert ,\Vert {-\hat{w}}\Vert \bigr\} }. $$

Consequently, $\{w_{n}\}$ is bounded, and so is $\{v_{n}\}$.

Let $T=2P_{S}-I$. From Proposition 2.2, one can know that the projection operator $P_{S}$ is monotone and nonexpansive, and $2P_{S}-I$ is nonexpansive.

Therefore,

$$\begin{aligned} w_{n+1} =&\frac{I+T}{2}\biggl[(1-\lambda_{n})w_{n}+ \lambda_{n}\biggl(1-\frac{\mu _{n}}{\lambda_{n}}G^{*}G\biggr)v_{n}\biggr] \\ =&\frac{I-\lambda_{n}}{2}w_{n}+\frac{\lambda_{n}}{2}\biggl(I- \frac{\mu_{n}}{\lambda _{n}}G^{*}G\biggr)v_{n}+\frac{T}{2}\biggl[(1- \lambda_{n})w_{n}+\lambda_{n}\biggl(I- \frac{\mu _{n}}{\lambda_{n}}G^{*}G\biggr)v_{n}\biggr], \end{aligned}$$

that is,

3.8 $$\begin{aligned} w_{n+1}=\frac{1-\lambda_{n}}{2}w_{n}+\frac{1+\lambda_{n}}{2}b_{n}, \end{aligned}$$

where $b_{n}=\frac{\lambda_{n}(I-\frac{\mu_{n}}{\lambda _{n}}G^{*}G)v_{n}+T[(1-\lambda_{n})w_{n}+\lambda_{n}(I-\frac{\mu_{n}}{\lambda _{n}}G^{*}G)v_{n}]}{1+\lambda_{n}}$.

Indeed,

3.9 $$\begin{aligned} & \Vert b_{n+1}-b_{n}\Vert \\ &\quad\leq \frac{\lambda_{n+1}}{1+\lambda_{n+1}}\biggl\Vert \biggl(I-\frac{\mu_{n+1}}{\lambda _{n+1}}G^{*}G \biggr)v_{n+1}-\biggl(I-\frac{\mu_{n}}{\lambda_{n}}G^{*}G\biggr)v_{n}\biggr\Vert +\biggl\vert \frac{\lambda _{n+1}}{1+\lambda_{n+1}}-\frac{\lambda_{n}}{1+\lambda_{n}}\biggr\vert \\ &\qquad{}\times \biggl\Vert \biggl(I-\frac{\mu_{n}}{\lambda_{n}}G^{*}G\biggr)v_{n}\biggr\Vert +\frac{T}{1+\lambda _{n+1}}\biggl\{ (1-\lambda_{n+1})w_{n+1}+ \lambda_{n+1}\biggl(I-\frac{\mu _{n+1}}{\lambda_{n+1}}G^{*}G\biggr)v_{n+1} \\ & \qquad{}-\biggl[(1-\lambda_{n})w_{n}+\lambda_{n} \biggl(I-\frac{\mu_{n}}{\lambda_{n}}G^{*}G\biggr)v_{n}\biggr]\biggr\} +\biggl\vert \frac{1}{1+\lambda_{n+1}}-\frac{1}{1+\lambda_{n}}\biggr\vert \\ & \qquad{}\times \biggl\Vert T\biggl[(1-\lambda_{n})w_{n}+ \lambda_{n}\biggl(I-\frac{\mu_{n}}{\lambda _{n}}G^{*}G\biggr)v_{n}\biggr] \biggr\Vert . \end{aligned}$$

For convenience, let $c_{n}=(I-\frac{\mu_{n}}{\lambda_{n}}G^{*}G)v_{n}$. By Lemma 2.5 in Shi *et al.* [1], it follows that $(I-\frac{\mu_{n}}{\lambda_{n}}G^{*}G)$ is nonexpansive and averaged. Hence,

3.10 $$\begin{aligned} & \Vert b_{n+1}-b_{n}\Vert \\ &\quad\leq\frac{\lambda_{n+1}}{1+\lambda_{n+1}}\Vert c_{n+1}-c_{n}\Vert +\biggl\vert \frac {\lambda_{n+1}}{1+\lambda_{n+1}}-\frac{\lambda_{n}}{1+\lambda_{n}}\biggr\vert \Vert c_{n} \Vert \\ & \qquad{}+\frac{T}{1+\lambda_{n+1}}\bigl\{ (1-\lambda_{n+1})w_{n+1}+\lambda _{n+1}c_{n+1}-\bigl[(1-\lambda_{n})w_{n}+ \lambda_{n}c_{n}\bigr]\bigr\} \\ & \qquad{}+\biggl\vert \frac{1}{1+\lambda_{n+1}}-\frac{1}{1+\lambda_{n}}\biggr\vert \bigl\Vert T\bigl[(1-\lambda _{n})w_{n}+\lambda_{n}c_{n} \bigr]\bigr\Vert \\ &\quad\leq\frac{\lambda_{n+1}}{1+\lambda_{n+1}}\Vert c_{n+1}-c_{n}\Vert +\biggl\vert \frac {\lambda_{n+1}}{1+\lambda_{n+1}}-\frac{\lambda_{n}}{1+\lambda_{n}}\biggr\vert \Vert c_{n} \Vert \\ &\qquad{}+\frac{1-\lambda_{n+1}}{1+\lambda_{n+1}}\Vert w_{n+1}-w_{n}\Vert + \frac{\lambda _{n+1}}{1+\lambda_{n+1}}\Vert c_{n+1}-c_{n}\Vert + \frac{\lambda_{n}-\lambda _{n+1}}{1+\lambda_{n+1}}\Vert w_{n}\Vert \\ & \qquad{}+\frac{\lambda_{n+1}-\lambda_{n}}{1+\lambda_{n+1}}\Vert c_{n}\Vert +\biggl\vert \frac {1}{1+\lambda_{n+1}}-\frac{1}{1+\lambda_{n}}\biggr\vert \bigl\Vert T\bigl[(1- \lambda_{n})w_{n}+\lambda _{n}c_{n}\bigr] \bigr\Vert . \end{aligned}$$

Moreover,

3.11 $$\begin{aligned} & \Vert c_{n+1}-c_{n}\Vert \\ &\quad=\biggl\Vert \biggl(I-\frac{\mu_{n+1}}{\lambda_{n+1}}G^{*}G\biggr)v_{n+1}- \biggl(I-\frac{\mu _{n}}{\lambda_{n}}G^{*}G\biggr)v_{n}\biggr\Vert \\ &\quad\leq \Vert v_{n+1}-v_{n}\Vert \\ &\quad=\bigl\Vert P_{S}\bigl[(1-\alpha_{n+1})w_{n+1}- \gamma_{n}G^{*}Gw_{n+1}\bigr]-P_{S}\bigl[(1-\alpha _{n})w_{n}-\gamma_{n}G^{*}Gw_{n}\bigr]\bigr\Vert \\ &\quad\leq\bigl\Vert \bigl(I-\gamma_{n+1}G^{*}G\bigr)w_{n+1}- \bigl(I-\gamma_{n+1}G^{*}G\bigr)w_{n}+(\gamma _{n}- \gamma_{n+1})G^{*}Gw_{n}\bigr\Vert \\ & \qquad{}+\alpha_{n+1}\Vert {-w_{n+1}}\Vert +\alpha_{n} \Vert w_{n}\Vert \\ &\quad\leq \Vert w_{n+1}-w_{n}\Vert +\vert \gamma_{n}-\gamma_{n+1}\vert \bigl\Vert G^{*}Gw_{n} \bigr\Vert +\alpha_{n+1}\Vert { -w_{n+1}}\Vert + \alpha_{n}\Vert w_{n}\Vert . \end{aligned}$$

Substituting (3.11) in (3.10), we infer that

3.12 $$\begin{aligned} & \Vert b_{n+1}-b_{n}\Vert \\ &\quad\leq\biggl\vert \frac{\lambda_{n+1}}{1+\lambda_{n+1}}-\frac{\lambda_{n}}{1+\lambda _{n}}\biggr\vert \Vert c_{n}\Vert +\frac{\lambda_{n}-\lambda_{n+1}}{1+\lambda_{n+1}}\Vert w_{n}\Vert + \frac {\lambda_{n+1}-\lambda_{n}}{1+\lambda_{n+1}}\Vert c_{n}\Vert \\ & \qquad{}+\Vert w_{n+1}-w_{n}\Vert +\biggl\vert \frac{1}{1+\lambda_{n+1}}-\frac{1}{1+\lambda_{n}}\biggr\vert \bigl\Vert T\bigl[(1- \lambda_{n})w_{n}+\lambda_{n}c_{n}\bigr] \bigr\Vert \\ & \qquad{}+\vert \gamma_{n}-\gamma_{n+1}\vert \Vert w_{n}\Vert +\alpha_{n+1}\Vert {-w_{n+1}}\Vert + \alpha_{n}\Vert w_{n}\Vert . \end{aligned}$$

By virtue of $\lim_{n\rightarrow\infty}(\lambda_{n+1}-\lambda_{n})=0$, it follows that $\lim_{n\rightarrow\infty} \vert \frac{\lambda_{n+1}}{1+\lambda_{n+1}}-\frac {\lambda_{n}}{1+\lambda_{n}}\vert =0$. Moreover, $\{w_{n}\}$ and $\{v_{n}\}$ are bounded, and so is $\{c_{n}\}$. Therefore, (3.12) reduces to

3.13 $$\begin{aligned} \lim\sup_{n\rightarrow\infty}\bigl(\Vert b_{n+1}-b_{n} \Vert -\Vert w_{n+1}-w_{n}\Vert \bigr)\leq {0}. \end{aligned}$$

Applying (3.13) and Lemma 2.4, we get

3.14 $$\begin{aligned} \lim_{n\rightarrow\infty} \Vert b_{n}-w_{n}\Vert =0. \end{aligned}$$

Combining (3.14) with (3.8), we obtain

$$\lim_{n\rightarrow\infty} \Vert x_{n+1}-x_{n}\Vert =0. $$

Using the convexity of the norm and (3.5), we deduce that

$$\begin{aligned} & \Vert w_{n+1}-\hat{w}\Vert ^{2} \\ &\quad\leq(1-\lambda_{n})\Vert w_{n}-\hat{w}\Vert ^{2}+\lambda_{n}\Vert v_{n}-\hat{w}\Vert ^{2} \\ &\quad\leq(1-\lambda_{n})\Vert w_{n}-\hat{w}\Vert ^{2}+\lambda_{n}\biggl\Vert -\alpha_{n}\hat {w}\\ &\qquad{}+(1-\alpha_{n})\biggl[w_{n}-\frac{\gamma_{n}}{1-\alpha_{n}}G^{*}Gw_{n}- \biggl(\hat{w}-\frac{\gamma_{n}}{1-\alpha_{n}}G^{*}G\hat{w}\biggr)\biggr]\biggr\Vert ^{2} \\ &\quad\leq(1-\lambda_{n})\Vert w_{n}-\hat{w}\Vert ^{2}+\lambda_{n}\alpha_{n}\Vert {-\hat{w}}\Vert ^{2}\\ &\qquad{}+(1-\alpha_{n})\lambda_{n}\biggl[\Vert w_{n}-\hat{w}\Vert ^{2} +\frac{\gamma_{n}}{1-\alpha_{n}}\biggl(\frac{\gamma_{n}}{1-\alpha_{n}}-\frac {2}{\rho(G^{*}G)}\biggr)\bigl\Vert G^{*}Gw_{n}-G^{*}G\hat{w}\bigr\Vert ^{2}\biggr] \\ &\quad\leq \Vert w_{n}-\hat{w}\Vert ^{2}+ \lambda_{n}\alpha_{n}\Vert {-\hat{w}}\Vert ^{2}+ \lambda_{n}\gamma _{n}\biggl(\frac{\gamma_{n}}{1-\alpha_{n}}- \frac{2}{\rho(G^{*}G)}\biggr)\bigl\Vert G^{*}Gw_{n}-G^{*}G\hat {w}\bigr\Vert ^{2}, \end{aligned}$$

which implies that

$$\begin{aligned} & \lambda_{n}\gamma_{n}\biggl(\frac{2}{\rho(G^{*}G)}- \frac{\gamma_{n}}{1-\alpha_{n}}\biggr)\bigl\Vert G^{*}Gw_{n}-G^{*}G\hat{w}\bigr\Vert ^{2} \\ &\quad\leq \Vert w_{n}-\hat{w}\Vert ^{2}-\Vert w_{n+1}-\hat{w}\Vert ^{2}+\lambda_{n} \alpha_{n}\Vert {-\hat {w}}\Vert ^{2} \\ &\quad \leq \Vert w_{n+1}-w_{n}\Vert \bigl(\Vert w_{n}-\hat{w}\Vert +\Vert w_{n+1}-\hat{w}\Vert \bigr)+ \lambda _{n}\alpha_{n}\Vert {-\hat{w}}\Vert ^{2}. \end{aligned}$$

Since $\lim\inf_{n\rightarrow\infty}\lambda_{n}\gamma_{n}(\frac{2}{\rho (G^{*}G)}-\frac{\gamma_{n}}{1-\alpha_{n}})>0$, $\lim_{n\rightarrow\infty }\alpha_{n}=0$ and $\lim_{n\rightarrow\infty} \Vert w_{n+1}-w_{n}\Vert =0$, we infer that

3.15 $$\begin{aligned} \lim_{n\rightarrow\infty}\bigl\Vert G^{*}Gw_{n}-G^{*}G\hat{w}\bigr\Vert =0. \end{aligned}$$

Applying Proposition 2.2 and the property of the projection $P_{S}$, one can easily show that

3.16 $$\begin{aligned} & \Vert v_{n}-\hat{w}\Vert ^{2} \\ &\quad=\bigl\Vert P_{S}\bigl[(1-\alpha_{n})w_{n}- \gamma_{n}G^{*}Gw_{n}\bigr]-P_{S}\bigl[\hat{w}-\gamma _{n}G^{*}G\hat{w}\bigr]\bigr\Vert ^{2} \\ &\quad\leq\bigl\langle {(1-\alpha_{n})w_{n}-\gamma_{n}G^{*}Gw_{n}- \bigl(\hat{w}-\gamma_{n}G^{*}G\hat {w}\bigr),v_{n}-\hat{w}}\bigr\rangle \\ &\quad=\frac{1}{2}\bigl\{ \bigl\Vert w_{n}-\gamma_{n}G^{*}Gw_{n}- \bigl(\hat{w}-\gamma_{n}G^{*}G\hat {w}\bigr)-\alpha_{n}w_{n} \bigr\Vert ^{2}+\Vert v_{n}-\hat{w}\Vert ^{2} \\ & \qquad{}-\bigl\Vert (1-\alpha_{n})w_{n}-\gamma_{n}G^{*}Gw_{n}- \bigl(\hat{w}-\gamma_{n}G^{*}G\hat {w}\bigr)-v_{n}+\hat{w}\bigr\Vert ^{2}\bigr\} \\ &\quad\leq\frac{1}{2}\bigl\{ \Vert w_{n}-\hat{w}\Vert ^{2}+2\alpha_{n}\Vert {-w_{n}}\Vert \bigl\Vert w_{n}-\gamma _{n}G^{*}Gw_{n}-\bigl(\hat{w}- \gamma_{n}G^{*}G\hat{w}\bigr)-\alpha_{n}w_{n}\bigr\Vert \\ & \qquad{}+\Vert v_{n}-\hat{w}\Vert ^{2}-\bigl\Vert w_{n}-v_{n}-\gamma_{n}G^{*}G(w_{n}- \hat{w})-\alpha_{n}w_{n}\bigr\Vert ^{2}\bigr\} \\ &\quad \leq\frac{1}{2}\bigl\{ \Vert w_{n}-\hat{w}\Vert ^{2}+\alpha_{n}M+\Vert v_{n}-\hat{w}\Vert ^{2}-\Vert w_{n}-v_{n}\Vert ^{2} \\ &\qquad{}+2 \gamma_{n}\bigl\langle {w_{n}-v_{n},G^{*}G(w_{n}- \hat{w})}\bigr\rangle \\ & \qquad{}+2\alpha_{n}\langle{w_{n},w_{n}-v_{n}} \rangle-\bigl\Vert \gamma_{n}G^{*}G(w_{n}-\hat {w})+ \alpha_{n}w_{n}\bigr\Vert ^{2}\bigr\} \\ &\quad\leq\frac{1}{2}\bigl\{ \Vert w_{n}-\hat{w}\Vert ^{2}+\alpha_{n}M+\Vert v_{n}-\hat{w}\Vert ^{2} \\ &\qquad{}-\Vert w_{n}-v_{n}\Vert ^{2}+2 \gamma_{n}\Vert w_{n}-v_{n}\Vert \bigl\Vert G^{*}G(w_{n}-\hat{w})\bigr\Vert \\ & \qquad{}+2\alpha_{n}\Vert w_{n}\Vert \Vert w_{n}-v_{n}\Vert \bigr\} \\ &\quad\leq \Vert w_{n}-\hat{w}\Vert ^{2}+ \alpha_{n}M-\Vert w_{n}-v_{n}\Vert ^{2}+4\gamma_{n}\Vert w_{n}-v_{n} \Vert \bigl\Vert G^{*}G(w_{n}-\hat{w})\bigr\Vert \\ & \qquad{}+4\alpha_{n}\Vert w_{n}\Vert \Vert w_{n}-v_{n}\Vert , \end{aligned}$$

where $M>0$ satisfies

$$M\geq{\sup_{k}\bigl\{ 2\Vert {-w_{n}}\Vert \bigl\Vert w_{n}-\gamma_{n}G^{*}Gw_{n}-\bigl(\hat{w}- \gamma_{n}G^{*}G\hat {w}\bigr)-\alpha_{n}w_{n}\bigr\Vert \bigr\} }. $$

From (3.5) and (3.16), we get

$$\begin{aligned} & \Vert w_{n+1}-\hat{w}\Vert ^{2} \\ &\quad\leq(1-\lambda_{n})\Vert w_{n}-\hat{w}\Vert ^{2}+\lambda_{n}\Vert v_{n}-\hat{w}\Vert ^{2} \\ &\quad\leq \Vert w_{n}-\hat{w}\Vert ^{2}- \lambda_{n}\Vert w_{n}-v_{n}\Vert ^{2}+\alpha_{n}M+4\gamma_{n}\Vert w_{n}-v_{n}\Vert \bigl\Vert \gamma_{n}G^{*}G(w_{n}- \hat{w})\bigr\Vert \\ & \qquad{}+4\alpha_{n}\Vert w_{n}\Vert \Vert w_{n}-v_{n}\Vert , \end{aligned}$$

which means that

$$\begin{aligned} \lambda_{n}\Vert w_{n}-v_{n}\Vert ^{2}\leq{}&\Vert w_{n+1}-w_{n}\Vert \bigl(\Vert w_{n}-\hat{w}\Vert +\Vert w_{n+1}-\hat{w}\Vert \bigr)+ \alpha_{n}M \\ & {}+4\gamma_{n}\Vert w_{n}-v_{n}\Vert \bigl\Vert \gamma_{n}G^{*}G(w_{n}-\hat{w})\bigr\Vert \\ & {}+4\alpha_{n}\Vert w_{n}\Vert \Vert w_{n}-v_{n}\Vert . \end{aligned}$$

Since $\lim_{n\rightarrow\infty}\alpha_{n}=0$, $\lim_{n\rightarrow\infty }\Vert w_{n+1}-w_{n}\Vert =0$ and $\lim_{n\rightarrow\infty} \Vert G^{*}Gw_{n}-G^{*}G\hat {w}\Vert =0$, we infer that

$$\lim_{n\rightarrow\infty} \Vert w_{n}-v_{n}\Vert =0. $$

Finally, we show that $w_{n}\rightarrow{\hat{w}}$. Using the property of the projection $P_{S}$, we derive

$$\begin{aligned} & \Vert v_{n}-\hat{w}\Vert ^{2} \\ &\quad=\biggl\Vert P_{S}\biggl[(1-\alpha_{n}) \biggl(w_{n}-\frac{\gamma_{n}}{1-\alpha _{n}}G^{*}Gw_{n}\biggr) \biggr]\\ &\qquad{}-P_{S}\biggl[\alpha_{n}\hat{w}+(1- \alpha_{n}) \biggl(\hat{w}-\frac{\gamma _{n}}{1-\alpha_{n}}G^{*}G\hat{w}\biggr)\biggr] \biggr\Vert ^{2} \\ &\quad\leq\biggl\langle (1-\alpha_{n}) \biggl(I-\frac{\gamma_{n}}{1-\alpha_{n}}G^{*}G \biggr) (w_{n}-\hat {w})-\alpha_{n}\hat{w},v_{n}- \hat{w}\biggr\rangle \\ &\quad\leq(1-\alpha_{n})\Vert w_{n}-\hat{w}\Vert \Vert v_{n}-\hat{w}\Vert +\alpha_{n}\langle\hat {w}, \hat{w}-v_{n}\rangle \\ &\quad\leq\frac{1-\alpha_{n}}{2}\bigl(\Vert w_{n}-\hat{w}\Vert ^{2}+\Vert v_{n}-\hat{w}\Vert ^{2}\bigr)+\alpha _{n}\langle\hat{w},\hat{w}-v_{n}\rangle, \end{aligned}$$

which equals

3.17 $$\begin{aligned} \Vert v_{n}-\hat{w}\Vert ^{2}\leq\frac{1-\alpha_{n}}{1+\alpha_{n}} \Vert w_{n}-\hat{w}\Vert ^{2}+\frac{2\alpha_{n}}{1-\alpha_{n}}\langle \hat{w},\hat{w}-v_{n}\rangle. \end{aligned}$$

It follows from (3.5) and (3.17) that

3.18 $$\begin{aligned} & \Vert w_{n+1}-\hat{w}\Vert ^{2} \\ &\quad\leq(1-\lambda_{n})\Vert w_{n}-\hat{w}\Vert ^{2}+\lambda_{n}\Vert v_{n}-\hat{w}\Vert ^{2} \\ &\quad\leq(1-\lambda_{n})\Vert w_{n}-\hat{w}\Vert ^{2}+\lambda_{n}\biggl\{ \frac{1-\alpha _{n}}{1+\alpha_{n}}\Vert w_{n}-\hat{w}\Vert ^{2}+\frac{2\alpha_{n}}{1-\alpha_{n}}\langle\hat {w}, \hat{w}-v_{n}\rangle\biggr\} \\ &\quad\leq\biggl(1-\frac{2\alpha_{n}\lambda_{n}}{1+\alpha_{n}}\biggr)\Vert w_{n}-\hat{w}\Vert ^{2}+\frac {2\alpha_{n}\lambda_{n}}{1-\alpha_{n}}\langle\hat{w},\hat{w}-v_{n}\rangle. \end{aligned}$$

Since $\frac{\gamma_{n}}{1-\alpha_{n}}\in{(0,\frac{2}{\rho(G^{*}G)})}$, we observe that $\alpha_{n}\in{(0,1-\frac{\gamma_{n}\rho(G^{*}G)}{2})}$, then

$$\frac{2\alpha_{n}\lambda_{n}}{1-\alpha_{n}}\in{\biggl(0,\frac{2\lambda_{n}(2-\gamma _{n}\rho(G^{*}G))}{\gamma_{n}\rho(G^{*}G)}\biggr)}, $$

that is to say,

$$\frac{2\alpha_{n}\lambda_{n}}{1-\alpha_{n}}\langle\hat{w},\hat{w}-v_{n}\rangle \leq \frac{2\lambda_{n}(2-\gamma_{n}\rho(G^{*}G))}{\gamma_{n}\rho(G^{*}G)}\langle \hat{w},\hat{w}-v_{n}\rangle. $$

By virtue of $\sum_{n=1}^{\infty}(\frac{\lambda_{n}}{\gamma_{n}})<\infty$, ${\gamma_{n}}\in{(0,\frac{2}{\rho(G^{*}G)})}$ and $\langle\hat{w},\hat {w}-v_{n}\rangle$ is bounded, we obtain $\sum_{n=1}^{\infty}(\frac{2\lambda _{n}(2-\gamma_{n}\rho(G^{*}G))}{\gamma_{n}\rho_{n}(G^{*}G)})\langle\hat{w},\hat {w}-v_{n}\rangle<\infty$, which implies that

$$\sum_{n=1}^{\infty}\frac{2\alpha_{n}\lambda_{n}}{1-\alpha_{n}}\langle \hat {w},\hat{w}-v_{n}\rangle\leq\infty. $$

Moreover,

3.19 $$\begin{aligned} \sum_{n=1}^{\infty}\frac{2\alpha_{n}\lambda_{n}}{1-\alpha_{n}}\langle \hat {w},\hat{w}-v_{n}\rangle=\sum_{n=1}^{\infty}\frac{2\alpha_{n}\lambda _{n}}{1+\alpha_{n}}\frac{1+\alpha_{n}}{1-\alpha_{n}}\langle\hat{w},\hat {w}-v_{n} \rangle, \end{aligned}$$

it follows that all the conditions of Lemma 2.5 are satisfied. Combining (3.18), (3.19) and Lemma 2.5, we can show that $w_{n}\rightarrow \hat{w}$. This completes the proof. □

## Numerical experiments

In this section, we provide several numerical results and compare them with Tian’s [21] algorithm (3.15)’ and Byrne’s [22] algorithm (1.2) to show the effectiveness of our proposed algorithm. Moreover, the sequence given by our algorithm in this paper has strong convergence for the multiple-sets split equality problem. The whole program was written in Wolfram Mathematica (version 9.0). All the numerical results were carried out on a personal Lenovo computer with Intel(R)Pentium(R) N3540 CPU 2.16 GHz and RAM 4.00 GB.

In the numerical results, $A=(a_{ij})_{P\times{N}}$, $B=(b_{ij})_{P\times{M}}$, where $a_{ij}\in[0,1]$, $b_{ij}\in[0,1]$ are all given randomly, $P, M, N$ are positive integers. The initial point $x_{0}=(1,1,\ldots,1)$, and $y_{0}=(0,0,\ldots,0)$, $\alpha_{n}=0.1$, $\lambda _{n}=0.1$, $\gamma_{n}=\frac{0.2}{\rho{(G^{*}G)}}$, $\mu_{n}=\frac{0.2}{\rho {(G^{*}G)}}$ in Theorem 3.1, $\rho_{1}^{n}=\rho_{2}^{n}=0.1$ in Tian’s (3.15)’ and $\gamma_{n}=0.01$ in Byrne’s (1.2). The termination condition is $\Vert Ax-By\Vert <\epsilon$. In Tables 1-4, the iterative steps and CPU are denoted by *n* and *t*, respectively.

**Table 1:** $\pmb{\epsilon=10^{-5}, P=3, M=3, N=3}$

	**n**	**t**
Sequence (3.3)	60	0.078
Tian’s (3.15)’	117	0.093
Byrne’s (1.2)	1,845	1.125

**Table 2:** $\pmb{\epsilon=10^{-10}, P=3, M=3, N=3}$

	**n**	**t**
Sequence (3.3)	120	0.156
Tian’s (3.15)	294	0.29
Byrne’s (1.2)	8,533	2.734

**Table 3:** $\pmb{\epsilon=10^{-5}, P=10, M=10, N=10}$

	**n**	**t**
Sequence (3.3)	63	0.093
Tian’s (3.15)	426	0.469
Byrne’s (1.2)	2,287	1.313

**Table 4:** $\pmb{\epsilon=10^{-10}, P=10, M=10, N=10}$

	**n**	**t**
Sequence (3.3)	123	0.25
Tian’s (3.15)	948	0.906
Byrne’s (1.2)	13,496	2.437
